# Classification of Parkinson’s disease by deep learning on midbrain MRI

**DOI:** 10.3389/fnagi.2024.1425095

**Published:** 2024-08-20

**Authors:** Thomas Welton, Septian Hartono, Weiling Lee, Peik Yen Teh, Wenlu Hou, Robert Chun Chen, Celeste Chen, Ee Wei Lim, Kumar M. Prakash, Louis C. S. Tan, Eng King Tan, Ling Ling Chan

**Affiliations:** ^1^National Neuroscience Institute (NNI), Singapore, Singapore; ^2^Duke-NUS Medical School, Singapore, Singapore; ^3^Singapore General Hospital, Singapore, Singapore

**Keywords:** machine learning, substantia nigra, Nigrosome-1, Parkinson’s disease, susceptibility, MRI, iron, neuromelanin

## Abstract

**Purpose:**

Susceptibility map weighted imaging (SMWI), based on quantitative susceptibility mapping (QSM), allows accurate nigrosome-1 (N1) evaluation and has been used to develop Parkinson’s disease (PD) deep learning (DL) classification algorithms. Neuromelanin-sensitive (NMS) MRI could improve automated quantitative N1 analysis by revealing neuromelanin content. This study aimed to compare classification performance of four approaches to PD diagnosis: (1) N1 quantitative “QSM-NMS” composite marker, (2) DL model for N1 morphological abnormality using SMWI (“Heuron IPD”), (3) DL model for N1 volume using SMWI (“Heuron NI”), and (4) N1 SMWI neuroradiological evaluation.

**Method:**

PD patients (*n* = 82; aged 65 ± 9 years; 68% male) and healthy-controls (*n* = 107; 66 ± 7 years; 48% male) underwent 3 T midbrain MRI with T2*-SWI multi-echo-GRE (for QSM and SMWI), and NMS-MRI. AUC was used to compare diagnostic performance. We tested for correlation of each imaging measure with clinical parameters (severity, duration and levodopa dosing) by Spearman-Rho or Kendall-Tao-Beta correlation.

**Results:**

Classification performance was excellent for the QSM-NMS composite marker (AUC = 0.94), N1 SMWI abnormality (AUC = 0.92), N1 SMWI volume (AUC = 0.90), and neuroradiologist (AUC = 0.98). Reasons for misclassification were right–left asymmetry, through-plane re-slicing, pulsation artefacts, and thin N1. In the two DL models, all 18/189 (9.5%) cases misclassified by Heuron IPD were controls with normal N1 volumes. We found significant correlation of the SN QSM-NMS composite measure with levodopa dosing (rho = −0.303, *p* = 0.006).

**Conclusion:**

Our data demonstrate excellent performance of a quantitative QSM-NMS marker and automated DL PD classification algorithms based on midbrain MRI, while suggesting potential further improvements. Clinical utility is supported but requires validation in earlier stage PD cohorts.

## 1 Introduction

Parkinson’s disease (PD) diagnosis is a major clinical challenge owing to its wide clinical and aetiological heterogeneity, its overlap with other entities, and the lack of reliable *in-vivo* biomarkers. The primary neuropathological hallmark of PD is the progressive loss of dopaminergic (DA) neurons in the iron-rich substantia nigra pars compacta (SNpc) (Dickson, 2012). Nigrosomes 1–5 are located within the SNpc, of which N1 is the largest, and the site of the most sensitive marker of PD pathology histologically (Dickson, 2012). When N1 degeneration occurs, neuromelanin is released and iron is deposited into the extra-cellular space.

Differences in iron within the SNpc can be detected on iron-sensitive MR sequences. On T2*-weighted or susceptibility weighted MRI (SWI), the normal SNpc appears hypointense and the normal N1 appears hyperintense, resulting in the “swallow-tail” sign (Schwarz et al., 2014), which has excellent classification performance for PD patients versus healthy controls (HC; sensitivity = 94.6%, specificity = 94.4%) (Mahlknecht et al., 2017). Neuromelanin differences can be detected on specialized neuromelanin-sensitive (NMS) fast spin-echo sequences (Blazejewska et al., 2013). However, both approaches require expert visual radiological assessment, and carry the risk of observer-dependent rater bias.

Susceptibility map weighted imaging (SMWI) avoids artifacts induced by phase, and has increased susceptibility contrast and SNR (Gho et al., 2014), allowing a more accurate N1 assessment. Radiological PD classification using SMWI has excellent performance (accuracy = 91.8–97.7%) (Liu et al., 2020; Sung et al., 2022). Deep learning (DL) approaches using SMWI can also identify N1 abnormality (Heuron IPD; Heuron Co., Ltd., Seoul, Republic of Korea) and diagnose PD with excellent classification performance (AUC = 0.95, Shin et al., 2021). However, this proprietary method has not been independently externally validated and the benefit of a DL approach that automates N1 volume quantification (Heuron NI; Heuron Co., Ltd., Seoul, Republic of Korea) (Jeong et al., 2022) is unknown. The original report on the validation of Heuron IPD was limited by its stringent selection of participants (e.g., according to the PET detection of nigrostriatal degeneration, or the appearance of the N1 on MRI) which, while helpful for model training, may have also inflated the validation AUC (Shin et al., 2021). Secondly, the control sample in the original report comprised individuals with drug-induced Parkinsonism, rather than normal healthy controls (Shin et al., 2021).

Other DL based tools for PD diagnostic classification using MRI have been described in the literature. Several studies have utilised conventional MRI of the cerebrum (e.g., T1-weighted, T2-weighted and FLAIR) to classify PD based on morphological differences, and have achieved accuracies of approximately 90–96% (Basnin et al., 2021; Dhinagar et al., 2021; Camacho et al., 2023, 2024; Mallik et al., 2023). However, atrophy of the cerebrum typically becomes prominent only in the later stages of disease progression, while earlier-stage neuropathology is located in the midbrain (Filippi et al., 2020; Pieperhoff et al., 2022). Fewer studies have investigated the used of midbrain neuropathology from MRI as input to DL algorithms (Huseyn, 2020). Secondly, since morphological changes are nonspecific, studies have utilised advanced MRI techniques such as QSM and NMS MRI of midbrain nuclei in DL tools for PD diagnosis, which are better able to detect PD-specific neuropathological processes such as iron and neuromelanin content (Shinde et al., 2019; Gaurav et al., 2022a; Wang et al., 2023; Chen et al., 2024).

In this study we evaluated the Heuron IPD and Heuron NI DL models on our database of PD patients with midbrain SMWI. We compared the N1 DL models against an iron-neuromelanin composite model to determine the value-add of NMS in PD diagnosis. We hypothesised that additional NMS provides integral data that would improve the classification performance compared to either N1 DL model alone. We hypothesised that the DL models would demonstrate classification performance of AUC > 0.9, comparable to an experienced neuroradiologist, and comparable to that previously reported by the model developers (Shin et al., 2021).

## 2 Methods

### 2.1 Patient population

We used MRI and clinical data from PD patients (OFF-medication state) and age-matched HC, who were recruited from clinics at our tertiary referral centre between 2019 and 2021. PD patients were diagnosed by four neurologists specializing in movement disorders (mean 17.8 years of experience) using the Movement Disorder Society Clinical Diagnostic Criteria for Parkinson’s disease (Postuma et al., 2015). Age-matched HC were recruited from the spouses of patients in hospital clinics, health screening and the community, and were absent of neurological conditions. We excluded subjects with MRI contraindications, claustrophobia, known neurological/psychiatric diagnosis other than PD, chronic debilitating medical conditions, or poor cognitive function that would hinder patients’ understanding of the study. This study was approved by the local ethics board and written informed consent was obtained from all participants.

### 2.2 MRI protocols

All MRI data were acquired on the same 3 T MRI system (Siemens Skyra, Erlangen, Germany). We acquired a 3D T2* SWI multi-echo gradient echo sequence with the following parameters: TR 48 ms, TE 13.77/26.39/39 ms, FA 20°, voxel size 0.5 × 0.5 × 1 mm^3^, 32 slices, duration: 4.15 min. An echo train length of 3 was determined to be an acceptable trade-off between SNR and clinically-feasible acquisition time.

We also acquired an NMS T1-weighted turbo spin echo sequence with the following parameters: TR 938 ms, TE 15 ms, voxel size 0.5 × 0.5 × 3 mm^3^, 13 slices, duration: 10.42 min. Both sequences were acquired in an oblique-coronal scan plane positioned perpendicular to the midbrain, to improve the N1 in-plane visualization.

### 2.3 QSM post-processing

Quantitative susceptibility in parts per billion (ppb) was computed from QSM using the STI Suite (Li et al., 2014). Brain extraction based on the magnitude images was performed using the FSL Brain Extraction Tool (BET2). Phase unwrapping and background field phase removal were performed using the HARPERELLA technique. Regularized k-space inverse filtering was performed on the processed phase images to generate the initial QSM images. An iterative k-space algorithm was used on the initial QSM images to yield the final mean susceptibility (iron deposition) map (Haacke et al., 2010; Barbosa et al., 2015).

### 2.4 SMWI post-processing

The SMWI images were reconstructed using the SMWI software (Seoul National University, Seoul, Republic of Korea) (Nam et al., 2017) from the multi-echo GRE images as follows: (1) the channel-combined magnitude images were created using the root sum of squares of the multi-channel magnitude images, (2) the channel-combined phase images were created as the mean after correcting for phase offsets of individual channels, (3) the magnitude images from each echo were combined by root sum of squares, (4) the phase images from each echo were unwrapped by Laplacian unwrapping and a frequency calculated per voxel, (5) the background field was removed from the frequency images, (6) the QSM images were reconstructed using the sparse linear equation and least-squares method, (7) a QSM mask was created based on a paramagnetic threshold value, (8) the SMWI was generated as the product of the combined magnitude image and the QSM mask.

### 2.5 Clinical severity measurements

All participants underwent a clinical motor assessment using the Movement Disorders Society Unified Parkinson’s Disease Rating Scale motor part (MDS-UPDRS-III) (Goetz et al., 2008), and the Hoehn and Yahr stage (H&Y) (Hoehn and Yahr, 1967). We also recorded the levodopa equivalent daily dose (LEDD) and disease duration (age at MRI minus age at diagnosis) for the PD group.

### 2.6 QSM-NMS composite heuristic measure

We formed a heuristic measure for PD classification by combining information from QSM and NMS scans with the following steps. (1) Blinded manual segmentation of the whole SN region using MRIcroGL (University of South Carolina, Columbia, SC) on QSM and NMS separately, on three consecutive slices by a neuroradiologist. The slices were selected by inspecting the images in cranio-caudal direction and identifying the first slice whereby the red nuclei were barely or no-longer visible, and the two inferior consecutive slices. (2) Thresholding of these SN volumes on QSM and NMS images separately, for low susceptibility and high neuromelanin content as previously described (Schwarz et al., 2011; Kim et al., 2018; Hartono et al., 2023). The threshold selected was that which maximized the difference between PD and control groups. (3) In the NMS images, calculation of the ratio of the 90^th^ to the 10^th^ percentile of the NMS signal within the SN mask defined the “NMS contrast range.” (4) The “QSM-NMS composite” score was defined as the product of the QSM and NMS-based volumes, and the NMS contrast range. We determined this formula based on the preliminary observations that PD patients had smaller SN volumes in QSM and NMS images, and more narrow ranges of contrast in NMS images. Thus, lower values indicated a higher likelihood of PD and *vice-versa*. We formed an aggregate measure by averaging the left and right-sided values, to increase the SNR and to reduce the multiple testing burden among the set of primary tests.

### 2.7 Deep learning models

**Figure 1:**
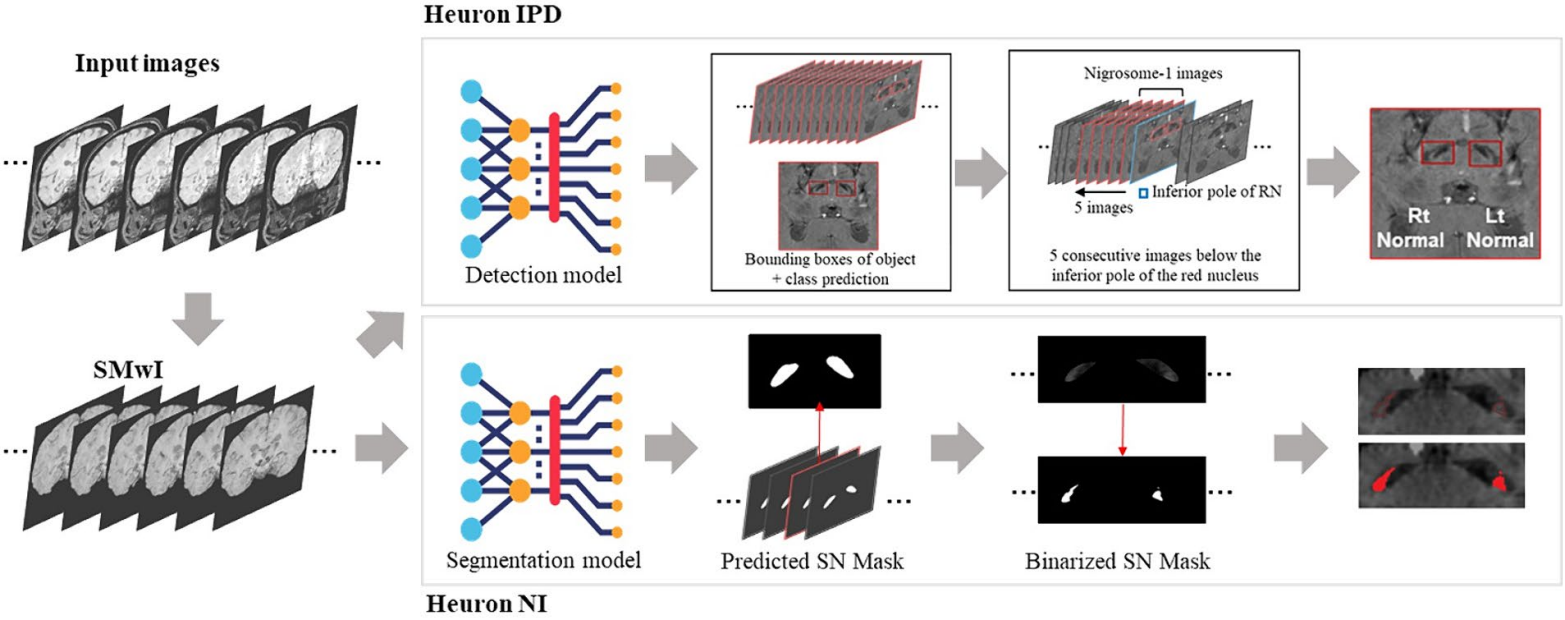
Summary of analysis pipelines of the two deep learning algorithms used in this study, and their differences. First, susceptibility-weighted images are used to create susceptibility-map-weighted images (SMWI), upon which the two models are run. For detection of nigrosome 1 morphological abnormalities, the first Heuron IPD model positions a bounding box to encompass the hypointense substantia nigra on each side. Then, using the slice containing the inferior-most pole of the red nucleus as reference and five consecutive slices inferior it, a classification is determined for either “normal” (N1 present) or “abnormal” (N1 lost) for each side. For the segmentation and volume quantification of nigrosome 1, Heuron NI first creates a mask of the whole SN within the bounding box in Heuron IPD. Within this, it applies a threshold to estimate the volume of nigrosome 1. (Note: neural network sub-parts are proprietary).

Two proprietary commercial DL models were provided to us (Figure 1; Heuron Co., Ltd., Seoul, Republic of Korea). In “Heuron IPD,” five slices were first automatically identified on the SMWI containing the N1 before detection of any abnormality (Shin et al., 2021). Abnormalities were detected using a convolutional deep neural network (CNN), YOLOv3 (Redmon and Farhadi, 2018), to detect morphological abnormality of the N1 region from the SMWI images. Heuron IPD returned a binary classification of “Normal” or “Abnormal” (Shin et al., 2021). “Heuron NI” automatically detected and segmented hyperintensities in the same N1-containing cuts on SMWI (Jeong et al., 2022), and returned the volume of the N1 in mm^3^. Heuron NI utilizes SparseInst for segmentation of the SN region (Cheng et al., 2022), and is based on a fully-convolutional encoder-decoder architecture, which includes backbone, context-encoder, and decoders to create instance activation maps. The model was trained using ResNet as the backbone, AdamW as the optimizer (with learning rate 5e-5) and a batch size of 16. The training of the model involved focal, dice, and binary cross entropy loss functions. Data augmentation was used to re-scale, and adjust brightness of the input data. Both programs provided left and right hemispheric results, which we analysed separately. For Heuron IPD, we also aggregated the left and right sided data by classifying subjects only as “Normal” if both the left and right N1 were “Normal,” and otherwise as “Abnormal.” For Heuron NI, we aggregated the left and right sided data by averaging the left and right volumes.

### 2.8 Neuroradiologist assessment

An experienced neuroradiologist (25 years) performed the assessment of SMWI while blinded to the subject status. Each side was rated as normal (clear visualization of N1), or abnormal (complete or suspected N1 loss). A subject was classified as normal when both sides were rated normal (Sung et al., 2022). Real time SMWI image reformatting was performed as needed (e.g., symmetry alignment, axial bicommisural planal or its orthogonal review) to improve the clarity for assessment as per routine clinical workflow.

### 2.9 Statistical analysis

Statistical analysis was performed using SPSS version 26 (IBM SPSS Statistics, IBM Corp, Armonk, NY). Continuous imaging measures were right-skewed in both groups and so we report the median and inter-quartile range statistics and used non-parametric tests. The clinical and demographic measures were approximately normally distributed so we report the mean and SD, and apply parametric tests. We performed ROC curve analysis to calculate the AUC. For the models with continuous outcomes (Heuron NI volume, QSM-NMS composite, QSM- and NMS-based volumes, NMS contrast range), we binarized the data using the Youden Index. We compared the AUCs of Heuron IPD, Heuron NI, neuroradiologist, and the QSM-NMS composite. Correlation of the continuous imaging measures in (1) each hemisphere separately and (2) averaged between hemispheres, with clinical parameters in both groups combined, and in the PD group only was performed using the Spearman rank correlation and Kendall Tao Beta. Multiple comparisons were controlled using the Bonferroni method (α = 0.05/40 = 0.00125).

## 3 Results

### 3.1 Sample characteristics

Our final sample comprised data from 189 participants including 82 PD (aged 65 ± 9 years; 68% male) and 107 HC (aged 66 ± 7 years; 48% male; Table 1). Patients had mild disease (MDS-UPDRS-III = 31 (20–38); H&Y stage = 2 (1–3); LEDD = 375 mg (250–564), disease duration = 4.8 (1.44–8.70) years). The PD and HC groups differed significantly on all quantitative imaging measures, both as whole groups (Table 1; *p* < 0.001) and when split by hemisphere (Table 2; *p* < 0.001). Heuron NI was unable to process one PD case due to a severe pulsation artifact, thus the sample size for Heuron NI is 188 (Supplementary Figures S1, S2, showing MRI images of the pulsation artefact and a flowchart of subject inclusion).

**Table 1:** Demographic and clinical information, and quantitative substantia nigra measurements for Parkinson’s disease and control groups, compared between groups.

	Parkinson’s disease, *n* = 82	Healthy controls, *n* = 107	Group difference, *p*-value
Age, years ^a^	65.00 (9.29)	65.51 (6.57)	0.656
Gender ^b^	Male = 56 (68%) Female = 26 (32%)	Male = 51 (48%) Female = 56 (52%)	0.005 **
MDS-UPDRS-III ^c^	31 (20–38)	2 (0–6)	<0.001 **
H&Y stage ^b^	0 = 0 (0%) 1 = 5 (6%) 2 = 68 (83%) 3 = 9 (11%)	0 = 107 (100%) 1 = 0 (0%) 2 = 0 (0%) 3 = 0 (0%)	<0.001 **
LEDD, mg ^c^	375 (250–564)	–	–
Disease duration, years between diagnosis and MRI ^c^	4.83 (1.44–8.70)	–	–
Heuron NI volume, mm^3^ ^c^	3.00 (1.35–5.44)	14.00 (8.94–20.00)	<0.001 **
QSM-based volume, mm^3^ ^c^	27.50 (15.50–45.00)	98.00 (56.00–142.00)	<0.001 **
NMS-based volume, mm^3^ ^c^	16.00 (6.00–27.00)	51.00 (25.00–108.00)	<0.001 **
NMS contrast range ^c^	1.139 (1.126–1.156)	1.173 (1.163–1.188)	<0.001 **

MDS-UPDRS-III, Movement Disorders Society Unified Parkinson Disease Rating Scale Part III; H&Y, Hoehn & Yahr; LEDD, levodopa equivalent daily dose; QSM, quantitative susceptibility mapping; NMS, neuromelanin-sensitive.**p < 0.01.

a Mean (standard deviation); groups compared by independent samples t-test.

b Frequency (percent); groups compared by Chi-square test.

c Median (inter-quartile range); groups compared by Mann–Whitney U test.

**Table 2:** Descriptive statistics for quantitative substantia nigra MRI measurements, split by hemisphere and compared between Parkinson’s disease and healthy control groups.

	Parkinson’s disease, *n* = 82	Healthy controls, *n* = 107	Group difference, *p*-value
Heuron NI volume, mm^3^ ^a^			
Left	2.75 (1.25–4.87)	14.62 (9.12–20.38)	<0.001 **
Right	2.38 (0.88–6.19)	11.88 (7.50–19.50)	<0.001 **
QSM-based volume, mm^3^ ^a^			
Left	25.00 (12.00–47.00)	95.00 (58.00–114.00)	<0.001 **
Right	27.50 (14.75–47.00)	93.00 (52.00–139.00)	<0.001 **
NMS-based volume, mm^3^ ^a^			
Left	23.00 (7.50–47.75)	106.00 (54.00–168.00)	<0.001 **
Right	16.50 (5.75–29.25)	51.00 (25.00–108.00)	<0.001 **
NMS contrast range ^a^			
Left	1.140 (1.126–1.156)	1.174 (1.163–1.192)	<0.001 **
Right	1.158 (1.136–1.172)	1.193 (1.175–1.211)	<0.001 **

QSM, quantitative susceptibility mapping; NMS, neuromelanin-sensitive.**p < 0.01.

a Median (inter-quartile range); groups compared by Mann–Whitney U test.

### 3.2 Classification performance

**Figure 2:**
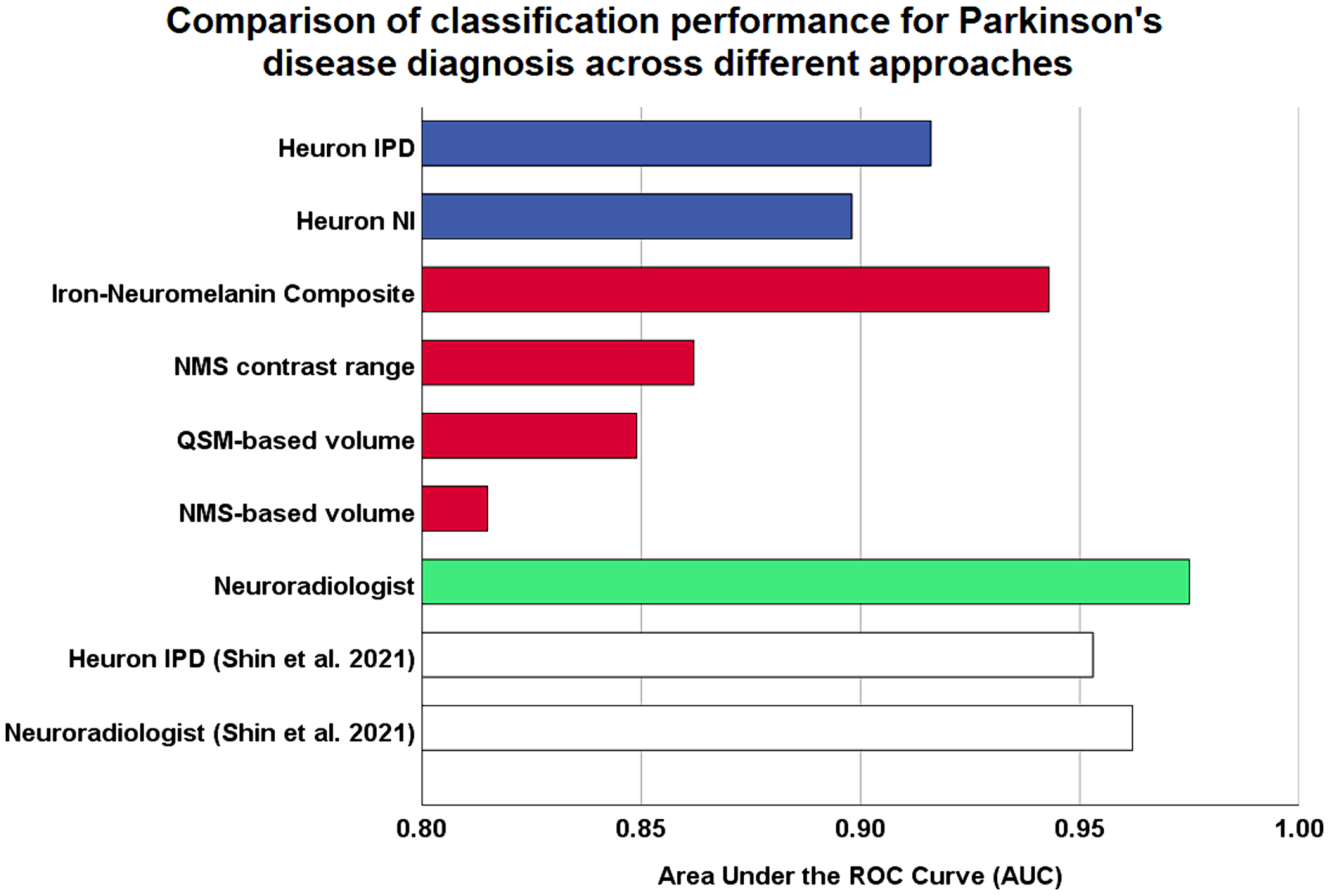
Comparison of model performances for Parkinson’s disease diagnostic classification using high resolution midbrain MRI. Coloured bars are based on original analysis performed in this study, while white bars represent classification performed by Shin et al. (2021), which reported the first validation study by the developers of their proprietary Heuron IPD deep learning model. Blue: fully-automatic deep learning models. Red: continuous imaging measures from manually-segmented substantia nigra. Green: visual radiological assessment of the nigrosome-1 sign using susceptibility map weighted imaging. ROC, receiver-operating characteristic.

The full classification performance results are summarized in Table 3. The QSM-NMS iron-neuromelanin composite measure showed an excellent classification performance (AUC = 0.94, accuracy = 89%, sensitivity = 94%, specificity = 86%; Figure 2), comparable to an experienced neuroradiologist (AUC = 0.98). The false positives (15/189; 7.9%) had smaller NMS- (*p* < 0.001) and QSM-based volumes (*p* = 0.018) than other HCs (Mann Whitney U tests). Accordingly, the false negatives (5/189; 2.6%) had larger Heuron NI (*p* = 0.021), NMS- (*p* = 0.008) and QSM-based volumes (*p* < 0.001), and iron-neuromelanin composite scores (*p* < 0.001; Mann–Whitney U tests).

**Table 3:** Results of the binary Parkinson’s disease versus healthy control classification by each model based on bilateral and single hemispheric findings.

Model	AUC	Accuracy (%)	Sensitivity (%)	Specificity (%)	Youden Index	Optimal cutoff
Heuron IPD	0.916	90.48	100.00	83.18	–	–
Left	0.912	90.48	97.56	85.05	–	–
Right	0.930	92.06	100.00	85.98	–	–
Heuron NI volume	0.898	84.57	83.95	85.05	0.69	6.88
Left	0.905	84.57	83.95	85.05	0.69	7.19
Right	0.846	78.72	81.48	76.64	0.58	7.19
QSM-NMS Composite	0.943	89.42	93.90	85.98	0.80	2178.62
Left	0.898	84.66	85.37	84.11	0.69	3297.83
Right	0.868	79.89	89.02	72.90	0.61	2110.04
NMS contrast range	0.862	80.95	84.15	78.50	0.63	1.1590
Left	0.837	80.42	79.27	81.31	0.60	1.1570
Right	0.832	78.31	76.83	79.44	0.55	1.1723
NMS-based volume	0.815	76.72	71.95	80.37	0.51	23.50
Left	0.832	78.31	79.27	77.57	0.56	53.50
Right	0.806	74.60	67.07	80.37	0.46	23.50
QSM-based volume	0.849	78.84	81.71	76.64	0.59	52.75
Left	0.856	80.42	76.83	83.18	0.60	49.00
Right	0.804	77.25	80.49	74.77	0.56	57.50
Neuroradiologist	0.975	97.35	98.78	96.26	–	–
Left	0.960	96.30	95.12	97.20	–	–
Right	0.958	96.30	93.90	98.13	–	–

AUC, area under the receiver operating characteristic curve; NMS, neuromelanin-sensitive; QSM, quantitative-susceptibility mapping.

Heuron IPD could also classify PD patients with excellent performance (AUC = 0.92, accuracy = 90% sensitivity = 100%, specificity = 83%). The 18/189 (9.5%) cases that were incorrectly classified (all false positives) had smaller left Heuron NI volumes (*p* = 0.010) and lower iron-neuromelanin composite scores (*p* = 0.039; Mann–Whitney U tests) than correctly-classified HCs.

Heuron NI (N1 volume) classified PD patients with moderate performance (AUC = 0.90, accuracy = 85%, sensitivity = 84%, specificity = 85%). There were both false positive (16/188; 8.5%) and false negative (13/188; 7.0%) classifications which, together, did not differ from the correctly classified cases in any demographic, clinical or imaging measure (Mann–Whitney U test; all *p* > 0.05). The false positives alone had smaller QSM-based volumes (*p* < 0.001) and lower iron-neuromelanin composite scores (*p* < 0.001) than the correctly-classified HCs (Mann–Whitney U tests). Conversely, the false negatives had larger QSM- (*p* < 0.001), and NMS-based volumes (*p* = 0.008), and iron-neuromelanin composite scores (*p* < 0.001) compared to the correctly classified PD patients (Mann–Whitney U tests). Thus, the falsely-classified subjects were outliers with regard to volume in their respective groups but could not be distinguished on demographic or clinical measures.

**Figure 3:**
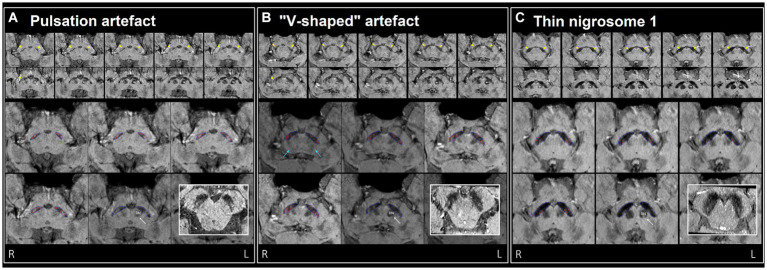
Examples of misclassification by DL model(s) in three healthy controls (false positives), **(A–C)** visually assessed as normal by neuroradiologist. Top two rows: ten consecutive 0.5 mm re-sliced caudo-cranial susceptibility map weighted (SMWI) output images from Heuron IPD, orientated perpendicular to the midbrain, showing the hyperintense nigrosome-1, N1 (yellow arrows) within the hypointense substantia nigra on cuts inferior to the red nucleus (marked “RN” on the left). Bottom two rows: magnified Heuron NI output images demonstrating hyperintensities outlined in red within the hypointense substantia nigra (blue) on four consecutive caudo-cranial cuts inferior to that containing the left red nucleus (RN) indicated by a double arrow. Bold inset image: intact N1 (“swallow-tail” sign) visualized as a dorsolateral hyperintensity in all three healthy controls on axial images reformatted parallel to the bi-commissural plane, providing a confirmatory alternative imaging perspective. **(A)** Pulsation artefacts from in-plane ambient cisternal arterial loops in this 47-year-old healthy male and slight right–left alignment asymmetry (tilted head - unequal red nuclei) could contribute to the “Abnormal” label of the right N1 on Heuron IPD. However, volume outputs on Heuron NI were normal. **(B)** 67-year-old healthy male with dark V-shaped (blue arrows) artefacts superimposed across the substantia nigra could impair N1 detection, and result in bilaterally “Abnormal” labels on Heuron IPD. Again, bilateral volume outputs on Heuron NI were normal. **(C)** Bilaterally skinny but distinct N1 in this 58-year-old female healthy control were labeled “Abnormal” by Heuron IPD. Volume outputs on Heuron NI were abnormally low; ideally, segmentation could have been automated on more inferior cuts after clearing both red nuclei.

As a benchmark, our experienced neuroradiologist performed a classification using the SMWI alone with excellent performance (AUC = 0.98, accuracy = 97%, sensitivity = 99%, specificity = 96%), with the most notable difference from the DL and quantitative approaches being the high specificity. A neuroradiologist visual *post-hoc* investigation of subjects misclassified by DL models identified (1) motion, cardiac pulsation (Supplementary Figure S1, showing pulsation artefact) and streaking artefacts, (2) bilaterally thin N1, (3) through-plane re-slicing which reduces signal and compounds aforesaid factors, (4) right–left alignment asymmetry from head tilt, (5) inappropriate slice selection for N1 segmentation, and (6) frequent overestimation of the N1 mask, as likely factors contributing to the false classifications of the DL models (Figure 3).

### 3.3 Hemispheric differences

The left hemisphere had consistently better performance than the right across all models except for Heuron IPD. The left–right discrepancy in AUCs for Heuron NI volume, NMS contrast range, NMS-based volume, QSM-based volume, iron-neuromelanin composite and neuroradiologist was 0.059, 0.005, 0.026, 0.052, 0.030 and 0.002, respectively. Our PD cohort was 100% right-handed, so we could not further evaluate the impact of handedness. The side of initial symptom onset for PD patients was left for *n* = 21, right for *n* = 49, symmetrical for *n* = 7 and unknown for *n* = 5. However, comparison of the left- and right-onset patients on the continuous imaging measures showed no significant effects (Mann–Whitney U test).

### 3.4 Correlation of imaging measures with clinical severity

Correlation of clinical motor symptom severity (MDS-UPDRS-III, H&Y stage) with the continuous imaging measures when both the PD and HC groups were included showed more severe disease correlating with smaller Heuron NI-, QSM- and NMS-based volumes and lower NMS contrast (Spearman ρ < −0.476, *p* < 6.75e^−12^).

**Figure 4:**
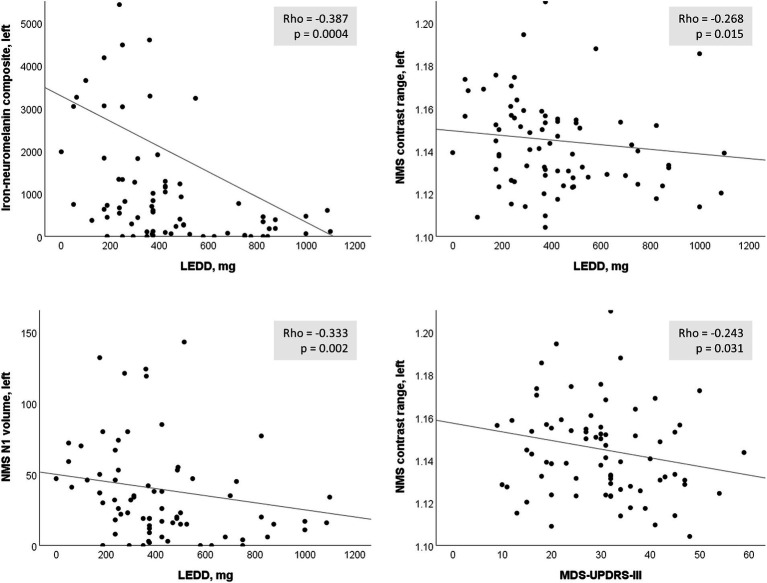
Significant left-sided correlations in the PD group between nigrosome-1 (N1) imaging measures and clinical parameters. LEDD, levodopa equivalent daily dose; NMS, neuromelanin-sensitive.

Based on the hemispheric differences we observed, we explored *post-hoc* correlations using individual hemispheric imaging measures in the PD group only, correcting for multiple comparisons using the Bonferroni method (α = 0.05/40 = 0.00125). Since we included the PD group only, we also tested for correlation with dosing (LEDD) and disease duration. This confirmed the predominant usefulness of left-sided imaging measures, with mostly left-sided results having *p* < 0.05 (Table 4; Figure 4). Correlation of the continuous imaging measures with severity (MDS-UPDRS-III, H&Y stage) and disease duration was weaker than that with levodopa dosing (LEDD), and was stronger for NMS-based than QSM-based measures. Correlation of the iron-neuromelanin composite and LEDD in the left hemisphere remained significant after multiple comparison correction (Spearman ρ = −0.303, *p* = 0.006).

**Table 4:** Correlation (correlation coefficient, *p*-value) of clinical severity, levodopa dosing and disease duration with quantitative substantia nigra measurements separately in each hemisphere in the Parkinson’s disease group only.

	MDS-UPDRS-III^a^	H&Y stage^b^	LEDD^a^	Disease duration^a^
Heuron NI volume				
Left	−0.117, 0.308	0.001, 0.995	−0.040, 0.722	0.005, 0.961
Right	−0.037, 0.745	0.126, 0.166	−0.016, 0.887	0.028, 0.803
QSM-based volume				
Left	−0.089, 0.437	−0.076, 0.397	−0.208, 0.062	−0.127, 0.260
Right	0.026, 0.819	0.141, 0.119	0.018, 0.874	0.106, 0.346
NMS-based volume				
Left	−0.149, 0.189	0.015, 0.868	−0.333, 0.002 *	−0.207, 0.064
Right	−0.061, 0.595	−0.035, 0.703	−0.202, 0.071	−0.020, 0.856
NMS contrast range				
Left	−0.243, 0.031 *	−0.027, 0.764	−0.268, 0.015 *	−0.215, 0.054
Right	−0.171, 0.133	−0.051, 0.572	−0.229, 0.040 *	−0.085, 0.449
Iron-neuromelanin composite				
Left	−0.230, 0.041 *	−0.030, 0.740	−0.387, 0.0004 **	−0.211, 0.059
Right	−0.134, 0.240	0.043, 0.632	−0.231, 0.038 *	−0.005, 0.964

MDS-UPDRS-III, Movement Disorders Society Unified Parkinson Disease Rating Scale Part III; H&Y, Hoehn & Yahr; LEDD, levodopa equivalent daily dose; QSM, quantitative-susceptibility mapping; NMS, neuromelanin-sensitive. **significant after multiple comparison correction, α < 0.00125.*p < 0.05.

a Spearman correlation, reported as ρ.

b Kendall Tao Beta correlation, reported as τ_b_.

Supplementary Table S1 shows the results of the correlation analyses between the left and right averaged continuous imaging measures and clinical severity for the PD group, which concur with the single-hemisphere results.

## 4 Discussion

The use of automated tools to supplement PD diagnosis is an ongoing important area of research. Recent progress has focused on classification by characterizing N1 using MRI contrasts sensitive to magnetic susceptibility (iron) and neuromelanin (Shin et al., 2021; Sung et al., 2021; Jokar et al., 2023). We compared classification performance of an iron-neuromelanin composite measure, Heuron IPD and Heuron NI (DL models based on SMWI) against that of an experienced neuroradiologist, to determine the potential value-add of NMS MRI and the independent external validity of the DL models. We demonstrated good value of a combined iron-neuromelanin (QSM-NMS) marker. We found excellent performance for each model, which was comparable to the radiologist. These results mark the first independent external validation of a method for automatic PD classification based on SMWI, supporting its efficacy, while suggesting further improvements that could be made.

An iron-neuromelanin marker had excellent classification performance (AUC = 0.94) exceeding that of either DL model alone, and similar to a recent study using an automated SN template approach (AUC = 0.95) (Jokar et al., 2023). Our method had better performance than other approaches to SN NMS classification; for example, automated NMS quantification (AUC = 0.83) (Gaurav et al., 2022b), and was similar to others using manual segmentation on QSM (AUC = 0.96) (Cheng et al., 2019). NMS-based MRI measures, including the iron-neuromelanin marker, had the strongest correlations with clinical severity and dosing than other quantitative imaging measures. Our marker was based on manual SN segmentation but, nonetheless, suggests a benefit to combining QSM and NMS in future DL approaches for PD diagnosis.

Heuron IPD also achieved excellent classification performance, confirming our hypothesis and supporting its external validity. The AUC of Heuron IPD was 0.92, while its counterparts in similar studies reported AUC = 0.95 (Shin et al., 2021; Jokar et al., 2023). These models were first published in 2021 (Shin et al., 2021) and were trained on a Korean cohort of PD patients and HCs but had not been independently externally validated. Our cohort is also East-Asian and both have similar disease severity (H&Y stage = 2). However, the classification performance could have been limited by our cohort’s younger age (65 versus 71 years). The training cohort was selected based on dopamine transporter DAT scan status and MRI-appearance of the N1 whereas ours was not, so our analysis may represent a more ecologically-valid scenario since DAT scan is not always available. A further important test will be to apply these methods to undiagnosed suspected PD and samples which have not been filtered based on neurodegeneration, N1 structure, or presence of other neurological or psychiatric conditions. Finally, while each model had comparable AUC to the neuroradiologist, the number of false positives was notably greater. This was common across models but, in general, classification should ideally err on the side of false positives rather than false negatives.

We found that the performance of Heuron NI (AUC = 0.90) was less than that of Heuron IPD. Misclassified cases generally showed motion or pulsation artefacts, intact but thin N1, right–left alignment asymmetry, or reduced signal secondary to re-slicing of the data through-plane, which could confound automated N1 detection. Additional steps to address these could improve classification. The left hemisphere was better for classification than the right hemisphere, and was the only hemisphere to have any significant correlation (after multiple comparison) with levodopa dosing. This may be explained by the tendency for symptoms to first occur on the dominant side (most often right), and thus predominance of left-sided SN neuropathology due to the decussation of cortico-pontine fibres. Concordance of expected side of disease pathology and imaging abnormality serves to validate the imaging approaches. The significant correlation with dosing, but not with severity, suggests structural brain alterations with medication use. Other studies identified relationships between some sub-scores of the MDS-UPDRS-III and manually-segmented N1/SN on T2-weighted MRI (Fu et al., 2016) but not the MDS-UPDRS-III as a whole.

Clinical demand in radiology for inclusion of high-resolution midbrain imaging in brain MRI orders for evaluation of Parkinsonism is on the rise with increasing availability, evidence of good diagnostic performance (Kuya et al., 2016; Sung et al., 2019), varied clinical presentations (Parkinson’s Disease Society of the United Kingdom, 2019) and complex co-morbidities in an aging population (Bloem et al., 2020). For example, an intact N1 and congruous quantitative SN measures in the presence of silent extra-nigral vascular pathology may be useful clinical decision support tools indicating that levodopa should be sparingly prescribed. The principal application for this technology in a clinical workflow is to distinguish patients who have overt N1-sign loss. This could facilitate filtering of cases for reporting between general or junior neuroradiologists and senior neuroradiologists based on case difficulty. Ideally, such tools should be incorporated into an automatic pipeline to not require additional steps, and should present the results directly on a clinical workstation, which requires regulatory approval (Choy et al., 2018). These could also be used as adjunct teaching tools to train radiologists unfamiliar with midbrain N1 assessment.

Future studies should apply SMWI-based DL models in earlier-stage or prodromal PD cohorts, and attempt to classify PD from other Parkinsonisms such as essential tremor (Perez Akly et al., 2019) which may be an early stage PD misdiagnosis (Welton et al., 2021). This approach should also be tested in non-East-Asian cohorts. A limitation is that SMWI requires a specific multi-echo acquisition (Nam et al., 2017) not part of routine clinical neuroimaging protocols. Technologist training is needed for accustomisation to anatomical landmarks for accurate 3D slab placement, as right–left symmetry alignment of the sub-nuclear structures on high resolution SMWI is sensitive to head tilt.

Strengths of our study include the independent, external nature of our validation of DL models. This is important because the cohort used for validation in the original report (Shin et al., 2021) included PD patients based on their PET or MRI status, which could have increased the reported AUC. Our comparisons to midbrain QSM and NMS MRI are original, and this enhanced neuroimaging evaluation of PD by yielding significant correlations with disease severity measures. This is noteworthy for its potential to objectively monitor disease progression compared to QSM-only approaches.

Our data show that automated algorithms, and an iron-NMS marker to augment radiologists’ decision-making for PD diagnosis are highly accurate. The DL models can be further improved by incorporation of NMS MRI information, identification of artefacts, combination of data across models, hemispheric information, automatic re-slicing, and further training on other cohorts. There is a potential role for this approach in future clinical workflows, especially to support non-expert radiologists.

## Data Availability

The data analyzed in this study is available from the corresponding author on reasonable request. Requests to access these datasets should be directed to LC, ling2chanSGH@gmail.com.
