# Supplementary material for: Classification of Parkinson’s disease by deep learning on midbrain MRI
Source: Front Aging Neurosci. 2024 Aug 20;16:1425095. doi: 10.3389/fnagi.2024.1425095 (PMC11369979; doi:10.3389/fnagi.2024.1425095)
Supplement: Supplementary file 1 [file Data_Sheet_1.docx]

**Supplemental Materials**


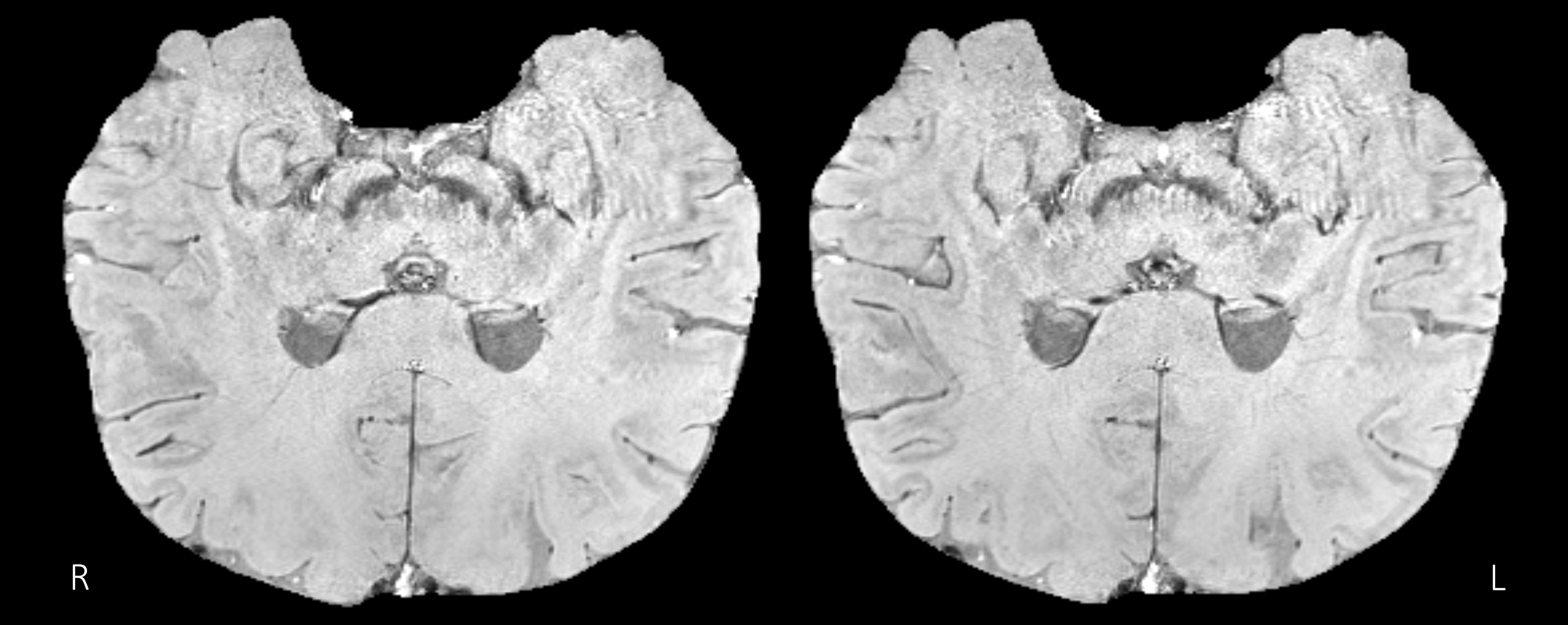


Figure S1. Consecutive susceptibility-map weighted images (SMWI) demonstrating severe arterial pulsation artefacts across the midbrain, causing misclassification or exclusion of the data from analysis.


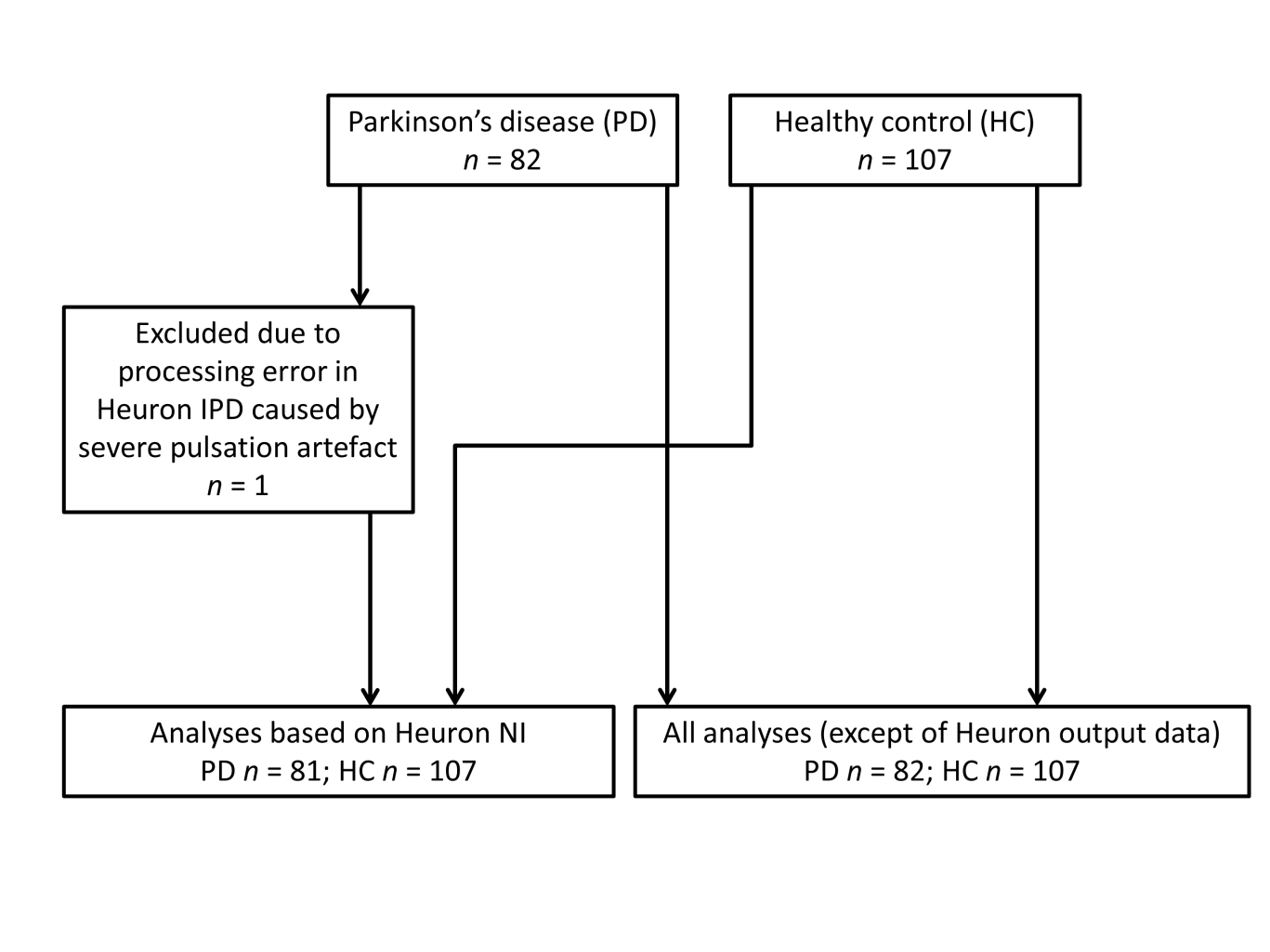


Figure S2. Flowchart of participants showing inclusion and exclusion. Only one participant was excluded due to severe pulsation artefacts precluding image processing by the Heuron IPD model.

Table S1. Correlation of clinical severity, dosing and disease duration with quantitative imaging measures (averaged between hemispheres) of the substantia nigra in the Parkinson’s disease group only (correlation coefficient, p-value).

|  | **MDS-UPDRS-III ^a^** | **H&Y stage ^b^** | **LEDD ^a^** | **Disease duration ^a^** |
| --- | --- | --- | --- | --- |
| **Heuron NI volume** | -0.048, 0.674 | 0.101, 0.265 | -0.026, 0.821 | 0.014, 0.903 |
| **QSM-based volume** | -0.024, 0.835 | 0.030, 0.740 | -0.117, 0.299 | -0.024, 0.831 |
| **NMS-based volume** | -0.085, 0.457 | -0.025, 0.782 | -0.256, 0.021 * | -0.048, 0.674 |
| **NMS contrast range** | -0.220, 0.051 | -0.010, 0.912 | -0.269, 0.015 * | -0.186, 0.096 |
| **Iron-neuromelanin composite** | -0.136, 0.234 | 0.060, 0.511 | -0.303, 0.006 * | -0.091, 0.421 |

MDS-UPDRS-III, Movement Disorders Society Unified Parkinson Disease Rating Scale Part III; H&Y, Hoehn & Yahr; LEDD, levodopa equivalent daily dose; QSM, quantitative-susceptibility mapping; NMS, neuromelanin-sensitive.
^a^ Spearman correlation, reported as ρ, p
^b^ Kendall Tao Beta correlation, reported as τ_b_, p
* p<0.05
